# Neurogenic Pelvic Floor Dysfunctions Across Neurological Disorders: Mechanisms, Phenotypes, and Precision Rehabilitation Pathways—A Narrative Review

**DOI:** 10.3390/jcm15135140

**Published:** 2026-07-01

**Authors:** Desirèe Latella, Chiara Scorza, Mirjam Bonanno, Andrea Calderone, Angelo Quartarone, Fabrizio Quattrini, Rocco Salvatore Calabrò

**Affiliations:** 1 IRCCS Centro Neurolesi Bonino Pulejo, 98124 Messina, Italy; desiree.latella@irccsme.it (D.L.); chiara_scorza@hotmail.it (C.S.); andrea.calderone@irccsme.it (A.C.); angelo.quartarone@irccsme.it (A.Q.); roccos.calabro@irccsme.it (R.S.C.); 2 Dipartimento di Scienze Cliniche Applicate e Biotecnologiche, Università Degli Studi Dell’Aquila, 67100 L’Aquila, Italy; fabrizio.quattrini@unicusano.it

**Keywords:** pelvic floor dysfunction, neurogenic bladder, neurorehabilitation, neurological disorders, urinary incontinence, pelvic floor muscle training, biofeedback, neuromodulation

## Abstract

**Background:** Pelvic floor dysfunction (PFD) is frequent in neurological disorders, but it is often approached as a secondary urological or gynecological problem rather than a functional rehabilitation target. Neurological disease can disturb cortical, pontine, spinal, sacral, autonomic, somatic, and sensory pathways that regulate bladder storage, voiding, bowel evacuation, sexual function, and pelvic pain modulation. **Methods:** This narrative review synthesized biomedical evidence identified through PubMed searches from database inception to 2 May 2026. Search concepts included neurogenic lower urinary tract dysfunction, urinary and bowel dysfunction, sexual dysfunction, pelvic pain, pelvic floor rehabilitation, biofeedback, electrical stimulation, neuromodulation, telerehabilitation, robotics, and major neurological disorders. The review was oriented according to the Scale for the Assessment of Narrative Review Articles (SANRA) and was not designed as a systematic review or meta-analysis. **Results:** Evidence from multiple sclerosis, stroke, Parkinson’s disease, Alzheimer’s disease and related dementias, spinal cord injury, and fibromyalgia or nociplastic pain syndromes supports a phenotype-based framework in which pelvic floor muscle training, bladder and bowel training, biofeedback, neuromuscular electrical stimulation, posterior tibial nerve stimulation, sacral neuromodulation, telerehabilitation, robotics, and multidisciplinary care are considered complementary rather than interchangeable strategies. **Conclusions:** PFD in neurological disorders may be more appropriately conceptualized as a multidimensional neurorehabilitation target. Effective care depends on disease-informed phenotyping, individualized rehabilitation goals, attention to cognition and adherence, and standardized outcome measurement. Future studies should test phenotype-specific pathways that integrate bladder, bowel, sexual, pain, participation, safety, and caregiver outcomes.

## 1. Introduction

Neurological disorders are a major and growing contributor to disability, mortality, and long-term care needs worldwide [1,2]. Their consequences often extend beyond motor, cognitive, sensory, or gait impairment and include less visible symptoms that strongly influence autonomy, dignity, participation, and quality of life. Pelvic floor dysfunction (PFD) belongs to this group of under-recognized complications. In this review, PFD is considered in a broad clinical sense, encompassing lower urinary tract symptoms (LUTS), urinary incontinence (UI), urgency, incomplete emptying, constipation, fecal incontinence, sexual dysfunction, dyspareunia, pelvic pain, and pelvic floor muscle-related dysfunction.

Pelvic floor symptoms are common in the general population, but neurological disorders add mechanisms that make assessment and treatment more complex. In non-neurological populations, symptoms often relate to age, parity, menopause, pelvic support, obesity, or comorbidity, whereas neurological populations may additionally present impaired voluntary control, altered sensation, autonomic dysfunction, cognitive impairment, medication burden, mobility limitation, reduced toileting access, and caregiver dependence [3,4,5,6,7]. This distinction provides the central rationale for the present review: pelvic floor complaints in neurological disorders should not be interpreted only as generic incontinence, constipation, sexual dysfunction, or pelvic pain, but as phenotype-specific problems shaped by neurological lesion pattern, functional context, and rehabilitation feasibility.

From a rehabilitation perspective, PFD should not be viewed only as a secondary urological or gynecological problem. Bladder, bowel, sexual, and pelvic pain symptoms may interfere with transfers, sleep, mood, intimacy, skin integrity, therapy adherence, caregiver routines, and community participation. They may also remain underreported because of stigma, embarrassment, cognitive or communication impairment, caregiver dependence, and limited clinician training. For this reason, neurological guidelines and clinical reviews increasingly support integrated assessment, including history, bladder diary, physical and neurological examination, postvoid residual assessment, urodynamic reasoning when indicated, renal-risk evaluation, medication review, and shared decision-making [8,9,10,11,12]. In clinical practice, this means that pelvic floor symptoms should be actively screened, interpreted according to neurological phenotype, and linked to rehabilitation goals rather than addressed only after complications occur. It also requires attention to feasibility, including fatigue, mobility limitations, communication barriers, cognitive status, privacy, cultural expectations, caregiver availability, digital access, and the patient’s ability to understand, practice, and maintain pelvic floor strategies over time within daily rehabilitation routines and home.

A phenotype-based approach is particularly important because the same diagnosis may generate different pelvic floor problems, whereas similar symptoms may require different interventions. MS may combine storage dysfunction, voiding impairment, fatigue, sensory disturbance, spasticity, and variable disease activity. Stroke may involve suprapontine disinhibition, aphasia, neglect, impaired mobility, altered awareness, and functional incontinence. Parkinson’s disease may combine urgency, nocturia, constipation, autonomic dysfunction, bradykinesia, falls risk, and cognitive vulnerability to anticholinergic medication. Alzheimer’s disease (AD) and related dementias may present functional incontinence, reduced symptom reporting, environmental barriers, caregiver-mediated toileting, and challenges concerning intimacy and consent. Spinal cord injury may involve detrusor overactivity, detrusor-sphincter dyssynergia, retention, catheter dependence, neurogenic bowel dysfunction, autonomic dysreflexia, sexual dysfunction, and renal safety concerns. Fibromyalgia and nociplastic pain syndromes are not included here as focal lesion-based neurological diseases. They are included as an adjacent mechanistic category because they are characterized by altered sensory processing, central sensitization, pain amplification, and frequent overlap with pelvic pain, urinary distress, bowel symptoms, sexual dysfunction, and pelvic floor guarding or overactivity. This category meets the conceptual inclusion criteria of the review because the synthesis maps mechanisms and rehabilitation phenotypes relevant to neurorehabilitation, not only structural central nervous system lesions. Its inclusion helps clarify when pelvic floor rehabilitation should prioritize down-training, graded exposure, pain education, relaxation, and multidisciplinary pain or sexual counseling rather than strengthening alone.

The aim of this narrative review is to synthesize current evidence on mechanisms, clinical phenotypes, conservative and technology-assisted rehabilitation strategies, outcome domains, and future precision pathways for neurogenic PFD in neurological and neurorehabilitation-relevant populations. The focus is on MS, stroke, Parkinson’s disease, AD and related dementias, spinal cord injury, and fibromyalgia or nociplastic pain syndromes because these conditions illustrate complementary vulnerability pathways, including demyelinating, suprapontine, basal ganglia-autonomic, cognitive-functional, spinal, sensory-processing, and pain-modulation mechanisms.

Compared with recent focused summaries of pelvic floor dysfunction in neurological disorders [13], this review adopts a broader precision-rehabilitation framework. It integrates neuro-urological mechanisms, disease-specific phenotypes, conservative and technology-assisted interventions, outcome standardization, caregiver feasibility, and implementation issues into a unified clinical reasoning pathway. This approach may help move the field from recognition of pelvic floor symptoms toward phenotype-informed rehabilitation planning, multidisciplinary care, and future trial design.

## 2. Materials and Methods

This narrative review was designed to provide a clinically oriented synthesis rather than a systematic review or meta-analysis, consistent with contemporary guidance on narrative review methodology and review typologies [14,15,16]. Accordingly, no formal systematic-review protocol, duplicate independent screening, PRISMA flow diagram, meta-analysis, or formal risk-of-bias/certainty assessment was performed. PubMed was searched from database inception to 2 May 2026 and the final search update was performed on 2 May 2026. PubMed was selected as the primary database because it was considered the most directly relevant biomedical source for peer-reviewed clinical, neuro-urological, neurological, rehabilitation, and pelvic-floor-related research. Searches combined terms related to pelvic floor dysfunction, neurogenic lower urinary tract dysfunction, urinary incontinence, bowel dysfunction, sexual dysfunction, pelvic pain, pelvic floor muscle training, biofeedback, electrical stimulation, neuromodulation, telerehabilitation, robotics, and neurological disorders. The full PubMed search strings, approximate search yields, and evidence-mapping logic are reported in Supplementary Table S1. The review considered peer-reviewed articles published in English and involving adult human populations. Eligible sources included randomized controlled trials, controlled clinical studies, prospective and retrospective observational studies, systematic reviews, meta-analyses, practice guidelines, terminology reports, and high-quality narrative reviews. Exclusion criteria were pediatric-only studies, pregnancy- or postpartum-only pelvic floor studies without neurological relevance, animal or preclinical studies, conference abstracts without full text, non-peer-reviewed material, and articles focused exclusively on surgical or pharmacological management without relevance to neuro-urological phenotyping, rehabilitation, conservative care, or multidisciplinary clinical pathways. Reference lists of relevant guidelines, systematic reviews, clinical trials, and mechanistic papers were also screened. The review was shaped according to the Scale for the Assessment of Narrative Review Articles (SANRA), which was used to guide narrative-review quality, topic justification, transparency of literature selection, balanced referencing, scientific reasoning, and presentation of data rather than to assess risk of bias in primary studies [14].

Eligible sources included randomized controlled trials, controlled clinical studies, prospective and retrospective observational studies, systematic reviews, meta-analyses, practice guidelines published as peer-reviewed articles, terminology reports, and high-quality narrative reviews. Studies were considered relevant when they addressed pelvic floor symptoms, LUTS, urinary storage or voiding dysfunction, bowel dysfunction, sexual dysfunction, pelvic pain, rehabilitation interventions, neuro-urological assessment, or functional outcomes in neurological or neurorehabilitation populations. Priority was given to evidence that informed the relationship between neurological mechanism, pelvic floor phenotype, rehabilitation feasibility, patient-reported outcomes, urodynamic variables, quality of life (QoL), adherence, cognitive considerations, or multidisciplinary implementation.

The neurological populations were selected to represent complementary mechanisms of pelvic floor vulnerability. MS, stroke, PD, AD and related dementias, and SCI were included because they illustrate demyelinating, suprapontine, basal ganglia-autonomic, cognitive-functional, and spinal pathway disruptions. Fibromyalgia and nociplastic pain syndromes were included as an adjacent mechanistic category because they illustrate how altered central pain processing and pelvic floor guarding may shape urinary, bowel, sexual, and pelvic pain phenotypes even in the absence of a focal structural neurological lesion. The synthesis was structured as an evidence-mapping framework organized around four complementary interpretive layers. The first layer addressed neuro-urological mechanisms, including supraspinal, pontine, spinal, autonomic, somatic, sensory, and pelvic floor pathways, and considered how neurological lesions or altered central processing may disturb bladder, bowel, sexual, and pelvic floor function. The second layer focused on disease-specific and cross-cutting clinical phenotypes, with separate attention to urinary, bowel, sexual, pelvic pain, cognitive, mobility, and caregiver-related domains across MS, stroke, PD, dementia, SCI, and fibromyalgia or nociplastic pain syndromes. The third layer examined conservative and technology-assisted rehabilitation approaches, including PFMT, bladder training, bowel training, biofeedback, NMES, PTNS, SNM, telerehabilitation, and robotic approaches. The fourth layer addressed implementation and future precision-neurorehabilitation pathways, including patient selection, phenotyping, cognition, adherence, caregiver involvement, safety, multidisciplinary ownership, feasibility, and standardized outcome measurement. These four layers guided the structure of the manuscript and the organization of tables and figures.

This approach allowed integration of heterogeneous evidence that would be difficult to pool quantitatively, particularly because the literature spans different diseases, outcomes, intervention doses, follow-up periods, and rehabilitation settings. To reduce subjectivity, conceptual relevance was operationalized as the presence of at least one of the following criteria: (1) description of neuro-urological, autonomic, spinal, supraspinal, sensory, pelvic floor, or pain-processing mechanisms relevant to pelvic floor dysfunction; (2) reporting of urinary, bowel, sexual, pelvic pain, quality-of-life, participation, adherence, or caregiver-related outcomes in neurological or neurorehabilitation populations; (3) evaluation of conservative, behavioral, pelvic floor, biofeedback, electrical stimulation, neuromodulatory, digital, telerehabilitation, robotic, or multidisciplinary interventions; (4) presentation of validated or clinically relevant assessment tools; or (5) provision of guideline, terminology, or expert-review evidence relevant to clinical reasoning. Sources were prioritized when they were recent, disease-specific, methodologically informative, or clinically relevant to phenotype-based rehabilitation planning.

These four layers guided the organization of the narrative synthesis, tables, and conceptual figures.

This evidence-mapping approach also considered feasibility variables that are often absent from efficacy trials. Cognitive status, fatigue, communication, mobility, caregiver availability, digital access, and tolerance of pelvic examination were treated as factors that can influence whether a rehabilitation strategy is realistic. These variables were not used as exclusion criteria for the synthesis. They were used to interpret how evidence might translate into neurological rehabilitation settings.

The latest available version of ChatGPT (OpenAI, San Francisco, CA, USA) was used only to support English-language editing, grammatical refinement, manuscript polishing, and visual/graphical refinement of figure layout. The figures were designed by the authors and graphically refined using the latest available version of ChatGPT (OpenAI, San Francisco, CA, USA), with final assembly and formatting performed by the authors. The tool was used only to support visual layout refinement and graphical organization. It was not used for data generation, statistical analysis, clinical interpretation, or evidence selection. All figure content, labels, captions, and scientific interpretations were critically reviewed, revised, and approved by the authors, who take full responsibility for the final manuscript.

## 3. Neuro-Urological and Pelvic Floor Control in Neurological Disorders

### 3.1. Central, Spinal, and Peripheral Control of Bladder, Bowel, and Sexual Function

**Figure 1:**
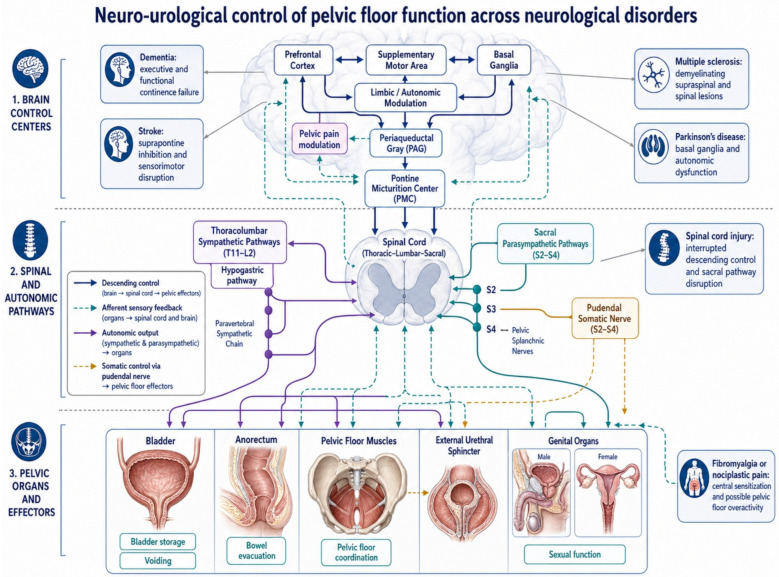
Neuro-urological control of pelvic floor function across neurological disorders. Legend: The figure was designed by the authors and graphically refined using the latest available version (5.5) of ChatGPT (OpenAI).

The lower urinary tract, anorectal system, pelvic floor muscles, and sexual organs are regulated through distributed neural networks rather than through a single reflex arc. Urodynamic assessment remains central when symptom patterns, renal risk, or treatment response require objective clarification. Good urodynamic practice and equipment standards help clinicians distinguish detrusor overactivity, impaired compliance, detrusor underactivity, bladder outlet obstruction, detrusor-sphincter dyssynergia, and stress leakage [17,18]. This is clinically important in neurological rehabilitation because symptoms do not always identify mechanisms. A patient may report urgency, yet the underlying picture may include sensory urgency, detrusor overactivity, incomplete emptying with overflow, or reduced toileting access. Urodynamic risk markers also help identify patients whose symptoms are mild, but whose bladder storage pressures may threaten the upper urinary tract [19,20,21]. Figure 1 summarizes the neuro-urological control network that links cortical, subcortical, pontine, spinal, sacral, peripheral, and muscular components.

Conceptual schematic showing the hierarchical cortical, subcortical, pontine, spinal, autonomic, and somatic pathways involved in bladder storage, voiding, bowel evacuation, pelvic floor coordination, sexual function, and pelvic pain modulation. Disease-specific callouts indicate how MS, stroke, Parkinson’s disease, dementia, spinal cord injury, and nociplastic pain syndromes may disrupt distinct levels of this integrated control system.

The mature micturition system depends on storage reflexes that maintain continence and on a coordinated switch to voiding when the social and physical context is appropriate [22]. The prefrontal cortex and related higher centers contribute to continence through planning, inhibition, attention, and behavioral control. The periaqueductal gray integrates bladder with afferent information with emotional and contextual signals. The pontine micturition center coordinates detrusor contraction with urethral sphincter relaxation. Sacral parasympathetic pathways drive detrusor contraction, thoracolumbar sympathetic pathways support storage, and pudendal somatic pathways control the external urethral sphincter and striated pelvic floor musculature [23,24,25].

Basal ganglia and dopaminergic pathways add another layer of regulation. The bladder is not merely a peripheral organ waiting for cortical permission. It sends afferent information that is processed through spinal, brainstem, and supraspinal circuits. Experimental and clinical work suggests that dopaminergic modulation can influence storage and voiding, which helps explain why urgency and OAB are frequent in PD and other parkinsonian syndromes [26,27,28]. Brainstem lesions, periaqueductal gray lesions, frontal lesions, and neurodegenerative processes may alter inhibitory tone or the ability to interpret bladder sensation. These mechanisms are not identical, but they converge clinically as urgency, frequency, nocturia, leakage, impaired emptying, or functional incontinence.

The pelvic floor muscles are anatomically peripheral but functionally central. They contribute to urethral closure, anal continence, pelvic organ support, sexual function, postural control, and breathing-related pressure regulation. Voluntary contraction depends on intact corticospinal and pudendal pathways, while reflex activity depends on sensory feedback and spinal integration. Clinical neurophysiology can evaluate pudendal nerve function, sacral reflexes, and pelvic floor muscle activation, although such tools are not routinely available in all rehabilitation settings [29]. Pelvic pain phenotypes add further complexity because pudendal neuralgia, pelvic myofascial pain, bladder pain, and central sensitization can overlap, making careful examination essential [30].

### 3.2. Mechanistic Links Between Neurological Injury, Pelvic Floor Dysfunction, and Rehabilitation Targets

SCI illustrates the mechanistic value of lesion level and completeness. Suprasacral lesions may disconnect pontine control from sacral reflexes, causing detrusor overactivity and dyssynergia. Sacral or peripheral lesions may cause detrusor underactivity, impaired sensation, and sphincter weakness. Autonomic dysfunction can transform bladder or bowel care into a cardiovascular safety issue, particularly when autonomic dysreflexia is triggered by bladder distension, catheter obstruction, bowel impaction, or urodynamic testing [31,32]. Same-session variability in urodynamics and cardiovascular responses also shows why single measurements must be interpreted within the broader clinical context [33].

Neurological mechanisms rarely operate in isolation. The Stockholm spinal cord uro study and contemporary SCI surveys indicate that urodynamic patterns, bladder management strategies, independence, and patient priorities interact over time [34,35]. The same is true in MS, where lesion dissemination, fatigue, sensory impairment, spasticity, cognition, and mobility can affect both symptom expression and rehabilitation feasibility. A mechanistic view therefore requires more than naming the disease. It requires identifying the dominant pathway disruption, the current pelvic floor phenotype, the patient’s functional reserve, and the likely barriers to training or behavioral change.

Rehabilitation targets can be mapped directly onto these mechanisms. PFMT aims to improve strength, endurance, coordination, timing, and awareness of pelvic floor contraction. Bladder training aims to modify urgency response, voiding intervals, fluid behavior, and learned avoidance. Bowel training aims to create predictable evacuation routines and reduce constipation or incontinence. Biofeedback enhances motor learning by converting otherwise hidden pelvic floor activity into visual or auditory information. NMES and peripheral stimulation can target afferent pathways, motor activation, or inhibitory reflexes, depending on protocol and population. A mechanistic framework does not guarantee success, but it prevents the common error of prescribing the same pelvic floor program to every neurological patient.

This section supports a practical proposition: neurogenic PFD is best understood as a disorder of interacting networks, not as failure of one muscle or one organ. The next step is to translate this framework into phenotypes that can be recognized during rehabilitation and outpatient neuro-urological care.

Pelvic floor rehabilitation is most coherent when the target is anatomically and physiologically plausible. Voluntary contraction is not equivalent to bladder inhibition, bowel evacuation, or pain reduction, even though these functions interact. Some patients can contract the pelvic floor but cannot suppress urgency because supraspinal inhibition is impaired. Others have high resting tones and poor relaxation during voiding or defecation. Patients with nociplastic pelvic pain may avoid contraction because tactile input or pelvic palpation amplifies symptoms. Assessment should therefore separate strength, endurance, coordination, relaxation, sensory awareness, timing, and symptom response.

Continence also depends on context. The nervous system must interpret bladder or rectal filling, decide whether voiding or evacuation is appropriate, maintain storage mechanisms, coordinate pelvic floor and sphincter activity, and permit timely access to toileting. Stroke may impair attention, planning, sensation, or mobility. PD may impair urgency inhibition, autonomic regulation, and timely movement. Dementia may impair awareness, initiation, and recognition of the toilet. SCI may interrupt descending coordination and create high-risk storage pressures. MS may produce variable combinations of sensory, motor, and autonomic pathway dysfunction.

Bowel and sexual symptoms share part of this architecture. Constipation, fecal incontinence, dyspareunia, erectile dysfunction, genital sensory loss, spasticity, and pelvic pain may involve the same sacral pathways, autonomic circuits, medications, behavioral routines, and caregiver constraints. Rehabilitation may act through strengthening, down-training, motor learning, sensory feedback, urgency inhibition, bowel routine optimization, or neuromodulation. Escalation to neuro-urology is not a failure of rehabilitation when unsafe storage, retention, recurrent infection, autonomic dysreflexia, or refractory symptoms dominate the phenotype.

A mechanism-based approach may also reduce therapeutic mismatch. Strengthening is reasonable when weakness, poor timing, or impaired urethral support dominate. Down-training is more coherent when pain, guarding, or dyssynergia dominates. Behavioral strategies may be preferable when urgency, delayed toileting, or cognitive routines drive symptoms. Specialist evaluation becomes more relevant when safety, retention, or upper tract risk is suspected. This logic links the neuro-urological circuit to practical rehabilitation decisions.

## 4. Clinical Phenotypes of Pelvic Floor Dysfunction Across Neurological Populations

### 4.1. Urinary, Bowel, Sexual, and Pelvic Pain Phenotypes

Clinical phenotyping begins with the symptom cluster rather than the diagnosis alone. Urinary phenotypes can be organized into storage, voiding, mixed, and catheter-related presentations. This classification is more clinically useful than listing symptoms individually, because each phenotype implies different rehabilitation priorities. Bowel phenotypes include constipation, prolonged evacuation, fecal incontinence, urgency, loss of evacuation awareness, and dependence on bowel routines. Sexual and pelvic pain phenotypes include reduced desire, arousal difficulties, orgasmic dysfunction, erectile dysfunction, vaginal dryness, dyspareunia, genital sensory loss, pelvic myofascial pain, and pain amplification.

**Figure 2:**
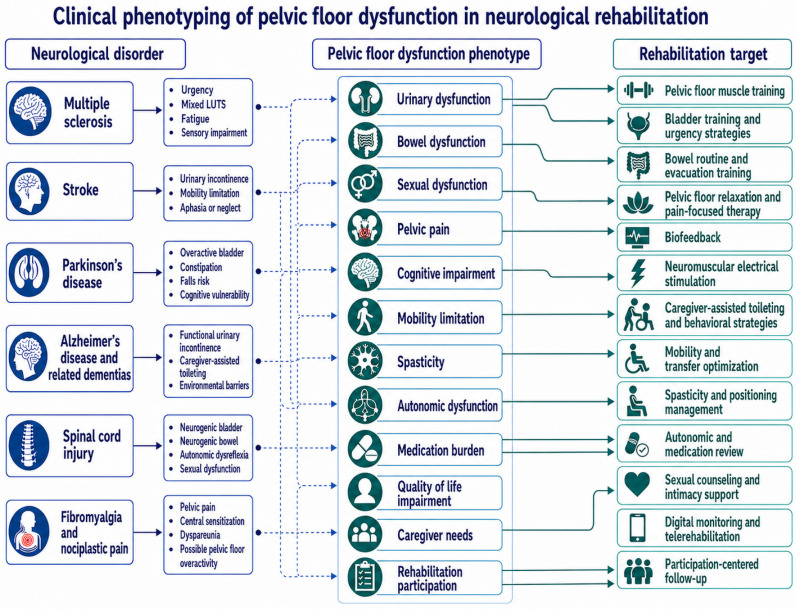
Clinical phenotyping of pelvic floor dysfunction in neurological rehabilitation. This conceptual phenotype map shows how neurological diagnosis informs the risk profile, whereas symptom phenotype helps define rehabilitation priorities. MS, stroke, Parkinson’s disease, AD and related dementias, spinal cord injury, and fibromyalgia or nociplastic pain are linked to representative phenotype clusters and to major rehabilitation targets, including pelvic floor muscle training, bladder and bowel rehabilitation, pain-focused therapy, biofeedback, neuromuscular electrical stimulation, caregiver-assisted strategies, mobility optimization, autonomic and medication review, sexual counseling, digital monitoring, and participation-centered follow-up. Legend: The figure was designed by the authors and graphically refined using the latest available version (5.5) of ChatGPT (OpenAI).

Table 1 maps major neurological conditions to dominant mechanisms, pelvic floor phenotypes, assessment priorities, and rehabilitation implications. Figure 2 presents a clinical phenotyping model that links urinary, bowel, sexual, pain, cognitive, mobility, autonomic, and QoL domains.

MS provides one of the clearest examples of mixed neurogenic pelvic floor involvement. LUTS are common, and systematic reviews confirm that storage symptoms, urgency, frequency, and UI are highly prevalent in people with MS [36,37]. Recent studies show that bladder symptoms can interact with fatigue, urinary QoL, depression, and well-being, supporting the view that LUTS are not minor inconveniences in this population [38,39,40]. A disease-specific interpretation is needed because MS may produce suprasacral dysfunction, spinal pathway involvement, impaired sensation, spasticity, fatigue-related participation barriers, and variable disease activity. Patients may also experience bowel and sexual symptoms, which are often under-addressed during routine neurological visits [74,75,76]. In MS, rehabilitation feasibility is likely to depend not only on the urinary phenotype but also on disability level, fatigue severity, sensory impairment, cognitive status, and disease course.

Stroke-related PFD is shaped by lesion location, acute disability, behavior control, mobility, sensory loss, and recovery stage. Contemporary global stroke data underscore the scale of the population that may require long-term rehabilitation [41,42]. Post-stroke LUTS are frequent, and urinary symptoms may occur during the acute stage, the rehabilitation phase, or chronic community living [43]. Newer evidence links post-stroke UI with behavior control deficits and OAB, while postvoid residual volume assessment can help identify patients who require closer monitoring after acute stroke [44,45]. Recovery of continence early after stroke appears prognostically meaningful, and persistent dysfunction may signal broader functional vulnerability [46]. Sensorimotor and cognitive impairments also affect the feasibility of PFMT because patients must perceive, isolate, and repeatedly activate the pelvic floor muscles [47]. In stroke, pelvic floor and continence management should be interpreted according to recovery stage. Early care may prioritize bladder scanning, scheduled toileting, transfer safety, and medication review, whereas later rehabilitation may allow more specific PFMT, urgency suppression, and confidence-building strategies.

PD illustrates the interaction between autonomic dysfunction, basal ganglia mechanisms, mobility, cognition, constipation, and falls at risk. Epidemiological evidence shows that PD is common in aging populations, and LUTS, UI, and urinary retention occur with variable prevalence across studies [48,49,50]. Urodynamic and autonomic profiles may help distinguish PD from atypical parkinsonian syndromes in selected cases [51]. Guidelines for bladder dysfunction in PD emphasize careful assessment, differentiation from other gait disorders, and individualized treatment [52]. Pharmacological options such as mirabegron have been investigated, and meta-analytic evidence continues to evaluate medication efficacy and safety [53,54]. From a rehabilitation perspective, the key question is whether behavioral and pelvic floor interventions can reduce urgency without worsening cognition, constipation, or orthostatic vulnerability.

### 4.2. Disease-Specific Patterns Across Neurological Disorders

Dementia and AD require different clinical logic. UI may reflect lower urinary tract pathology, but it may also arise from cognitive impairment, impaired initiation, reduced motivation, apraxia, environmental barriers, poor mobility, or inability to communicate urgency. Dementia prevalence studies and cohort analyses show that continence care is a major primary care and long-term care issue in older adults [55,56,57]. AD-specific studies suggest associations between UI, dementia severity, activities of daily living, and urodynamic diagnoses [58,59]. Lower urinary tract dysfunction in dementia must also be interpreted in the context of anticholinergic exposure, polypharmacy, and caregiver capacity [60]. Rehabilitation may therefore emphasize scheduled toileting, prompted voiding, environmental adaptation, caregiver education, and simplified pelvic floor cues rather than complex independent exercise programs.

SCI is the neurological condition in which pelvic organ dysfunction is often most overt, yet it still requires careful phenotyping. Global epidemiology confirms that SCI affects younger and older adults and creates long-term motor, sensory, autonomic, bowel, bladder, and sexual consequences [35]. Prediction studies and longitudinal cohorts show that bladder outcomes after traumatic SCI vary with neurological level, completeness, and recovery trajectory [61,62]. Bowel dysfunction is common in patients with neurogenic bladder, and meta-analytic evidence shows that neurogenic bowel dysfunction can profoundly reduce QoL in SCI [63,64]. Sexual functioning has also been recognized as a key recovery priority, and recent mixed-methods work highlights how sexuality is shaped by physical function, self-image, relationships, sensation, and access to counseling [65,66]. In SCI, pelvic floor rehabilitation should be embedded within a broader bladder and bowel safety plan, because continence goals may coexist with catheter routines, upper tract surveillance, autonomic dysreflexia prevention, bowel program optimization, and sexual rehabilitation.

Fibromyalgia and nociplastic pain syndromes complicate the boundaries of a neurogenic review because they are not defined by focal structural lesions. They are nevertheless relevant because altered sensory processing, possible pelvic floor overactivity or guarding, pelvic pain, urinary distress, bowel symptoms, and sexual dysfunction often coexist. Systematic review evidence supports an association between fibromyalgia and pelvic floor disorders [67]. Case–control and cross-sectional studies show higher pelvic floor symptom burden, urinary distress, sexual dysfunction, and pelvic floor dysfunction in women with fibromyalgia compared with controls [68,69,70]. Recent studies and reviews confirm the relevance of sexual dysfunction in fibromyalgia and support the need to view pelvic floor complaints through a pain-processing lens [71,72]. Nociplastic pain has also been associated with greater pelvic pain severity and pelvic myofascial pain, which supports the need for graded, non-threatening rehabilitation strategies rather than purely strengthening-oriented protocols [73].

These phenotypes are not mutually exclusive. An older woman with MS may have urgency from neurogenic detrusor overactivity, stress leakage from pelvic floor weakness, constipation from immobility, sexual pain from possible pelvic floor overactivity or guarding, and fatigue that limits adherence. A man with PD may have urgency, nocturia, constipation, erectile dysfunction, and cognitive vulnerability to anticholinergic medication. A patient with SCI may prioritize bowel independence or sexual counseling over a urodynamic variable. The phenotype therefore determines the rehabilitation question. Is the main target inhibition of urgency, support of urethral closure, coordination during voiding, predictable bowel care, reduction of pelvic pain, safe catheter independence, or participation in daily life?

This clinical framing prepares the transition to rehabilitation. Once the phenotype is defined, the next task is to match the intervention to the mechanism, the patient’s neurological capacity, and the implementation context.

Phenotyping should begin with concrete symptom patterns rather than broad labels. Leakage during urgency, transfers, coughing, delayed toileting, nocturnal waking, catheterization, or unrecognized episodes may point to different mechanisms. Delayed toileting may reflect stress leakage, urgency, spasticity, impaired trunk control, or poor anticipatory pelvic floor activation. Persistent dampness without awareness may reflect cognitive impairment, sensory loss, overflow, or functional incontinence. A single term such as UI is therefore insufficient for rehabilitation planning.

Bowel, sexual, and pain phenotyping require similar precision. Constipation may refer to infrequent stool, hard stool, prolonged evacuation, straining, incomplete emptying, digital assistance, unpredictable accidents, or excessive bowel care time. Sexual assessment should address desire, arousal, erection, lubrication, orgasm, dyspareunia, genital sensation, spasticity during intimacy, fear of leakage, body image, and partner communication. Pelvic pain assessment should consider myofascial tenderness, overactivity or guarding, pudendal neuralgia, bladder pain, trauma history, mood, sleep, and central sensitization features.

Disease-specific patterns help clinicians anticipate presentations, but they should not replace individual assessment. MS, stroke, PD, dementia, SCI, and fibromyalgia each create different vulnerability profiles. Implementation variables are equally relevant. Toilet distance, clothing, transfers, hand function, catheter access, privacy, caregiver schedules, health literacy, digital access, and cultural beliefs can determine whether a theoretically appropriate intervention is feasible in daily life. These details are not secondary to evidence. They are the conditions under which evidence becomes care.

## 5. Conservative and Technology-Assisted Rehabilitation Strategies

### 5.1. Pelvic Floor Muscle Training, Bladder Training, Bowel Training, Biofeedback, and Behavioral Therapy

Pelvic floor muscle training remains the most recognizable conservative intervention for UI, and high-quality evidence supports its role in women with stress, urgency, and mixed UI in non-neurological populations [77]. More recent Cochrane evidence comparing PFMT approaches highlights that dose, supervision, exercise type, and delivery format matter, although the optimal protocol is not identical for every patient [78]. Telerehabilitation meta-analysis suggests that remote PFMT can be feasible and effective in women with UI, which has important implications for neurological patients who face mobility, transport, fatigue, or access barriers [79]. Importantly, evidence for PFMT should be interpreted according to population and phenotype. The strongest direct evidence derives from women with non-neurogenic stress, urgency, or mixed UI, while neurological evidence is currently strongest for MS and selected post-stroke populations [79,80]. In SCI, PFMT is more plausibly applicable when incomplete pathways and sufficient sensory-motor control remain. In PD, dementia, and fibromyalgia/nociplastic pain syndromes, PFMT should be considered mainly as phenotype-guided clinical extrapolation unless disease-specific evidence and patient prerequisites support its use. These distinctions support PFMT as a core rehabilitation tool, but they do not justify treating it as a universal intervention independent of neurological phenotype.

Evidence specific to MS supports a cautious but optimistic view. Systematic review and meta-analysis data indicate that PFMT may improve LUTD in MS, although sample sizes, protocols, and outcomes remain heterogeneous [80]. Post-stroke evidence also supports potential benefits, with meta-analytic and systematic review findings suggesting improvements in UI and pelvic floor muscle function in selected stroke patients [81,82]. A foundational randomized trial in women after stroke showed that PFMT can be effective when patients are able to participate in training and when the intervention is delivered with appropriate supervision [83]. The clinical implication is not that every stroke or MS patient should receive the same PFMT protocol. The implication is that PFMT should be offered after assessment of awareness, contraction ability, cognition, fatigue, spasticity, and goals.

Telerehabilitation has moved from convenience to a strategic delivery model. Randomized and feasibility studies in MS show that remotely delivered PFMT can reduce barriers to specialized pelvic floor physiotherapy and may improve urinary symptoms, pelvic floor function, and patient engagement [84,85,86]. Remote delivery can also increase privacy for patients who might avoid in-person pelvic floor care. In PD, telerehabilitation may support behavioral coaching, bladder diaries, reminders, adherence monitoring, and access for patients with mobility limitations, but it requires caution when bradykinesia, falls risk, cognitive impairment, autonomic symptoms, constipation, or medication changes affect safety. In SCI, remote follow-up may support diary review, bladder and bowel routine coaching, education, and monitoring of access barriers, but it should not replace in-person neuro-urological assessment when high-risk NLUTD, autonomic dysreflexia, catheter problems, renal safety concerns, pressure injury risk, or complex bladder management are present. In AD and related dementias, telerehabilitation is rarely patient-directed and should usually be caregiver-mediated; potential uses include caregiver training, prompted voiding routines, environmental modification, and simplified monitoring, whereas limitations include consent capacity, cognitive impairment, digital exclusion, caregiver burden, privacy, and safety. Remote PFMT should not be reduced to unsupervised exercise videos. It is most defensible when it includes assessment, individualized progression, symptom diaries, feedback, adherence monitoring, and escalation criteria.

Adjunctive neuromodulatory strategies are being explored to enhance pelvic floor rehabilitation. A randomized clinical trial in women with MS suggested that anodal transcranial direct current stimulation combined with PFMT may enhance outcomes, which raises the possibility that cortical excitability and pelvic floor motor learning may be linked in selected patients [87]. Earlier randomized work comparing PFMT, electromyographic biofeedback, and NMES in MS also supports a multimodal model, particularly when isolated voluntary contraction is difficult or when feedback improves learning [88,89]. Controlled trials of pelvic floor programs and exercise in MS further suggest that rehabilitation can influence LUTS beyond the pelvic floor muscle itself, possibly through improved coordination, physical conditioning, and symptom self-management [90,91].

Behavioral therapy is especially relevant for PD because urgency suppression, bladder training, fluid strategies, toileting planning, and pelvic floor contractions can be adapted to motor and non-motor symptoms. A randomized clinical trial showed that behavioral therapy can improve urinary symptoms in PD [92]. Another randomized trial evaluating bladder training for urinary tract symptoms in PD supports the feasibility of structured non-pharmacological management [93]. These interventions are important because antimuscarinic therapy may be poorly tolerated in patients with constipation, cognitive vulnerability, or polypharmacy. Behavioral rehabilitation does not replace pharmacology in every case, but it provides a low-risk first-line or adjunct pathway when patients can learn and apply the strategy.

### 5.2. Electrical Stimulation, Neuromodulation, Telerehabilitation, Digital Monitoring, Robotics, and Integrated Rehabilitation Pathways

Electrical stimulation and peripheral neuromodulation broadens therapeutic opportunities. A systematic review of transcutaneous electrical nerve stimulation for neurogenic LUTD indicated potential benefits but also highlighted variability in protocols and evidence of quality [94]. A broader systematic review of nonsurgical, minimally invasive, or noninvasive therapies for UI due to neurogenic bladder supports the promise of conservative technologies while emphasizing heterogeneity [95]. Network meta-analysis data on neurogenic detrusor overactivity suggest that oral medications, onabotulinumtoxin A, and tibial nerve stimulation should be compared not only by efficacy but also by safety, invasiveness, and patient burden [96]. Peripheral electrical nerve stimulation meta-analysis reinforces that urodynamic and diary outcomes can improve, but standardized stimulation parameters remain needed [97].

SCI provides a strong rationale for neuromodulation research because bladder, bowel, sexual, and autonomic circuits are disrupted by lesion level and completeness. Transcutaneous electrical stimulation after SCI has been evaluated in systematic review and meta-analysis, with findings that support further trials but do not yet define a universal protocol [98]. External NMES can also influence pelvic floor muscle strength, urinary symptoms, QoL, sexual function, and patient satisfaction in women with urgency UI, although extrapolation to neurological populations should be careful [99]. Incomplete SCI trials suggest that PFMT and intravaginal electrical stimulation may improve UI in women who retain some sensory-motor capacity [100].

Magnetic stimulation and cortical approaches are emerging, particularly for neurogenic bladder after stroke and MS. A systematic review of transcranial magnetic stimulation and bladder function provides the mechanistic rationale for modulating supraspinal and spinal pathways [101]. Trials comparing cortical versus sacral repetitive magnetic stimulation in MS, and newer randomized work in post-stroke neurogenic bladder, suggest that noninvasive brain or sacral stimulation could become part of selected rehabilitation pathways [102,103]. Recent trials comparing transcranial magnetic stimulation with biofeedback and pilot studies of neuromodulation in MS show why this field is moving quickly but still requires replication, rigorous sham control, standardized endpoints, and longer follow-up [104,105].

Technology-assisted rehabilitation should be integrated rather than simply added. Bladder function training combined with pelvic floor biofeedback electrical stimulation has shown benefits in neurogenic bladder and urodynamics [106]. Transcutaneous electrical tibial nerve stimulation has been tested in parkinsonian syndromes, and neuromodulation for storage of LUTS in PD has been reviewed systematically [107,108]. Phase II data on transcutaneous tibial nerve stimulation for OAB symptoms in PD, randomized evidence on PTNS in MS, and systematic evidence on PTNS and other neuromodulations in MS support a stepwise pathway for selected patients [109,110,111]. Non-invasive neuromodulation for bowel, bladder, and sexual restoration after SCI also suggests that pelvic organ rehabilitation should be considered a multi-system target rather than a bladder-only intervention [112]. Table 2 summarizes conservative, behavioral, technology-assisted, neuromodulatory, and multidisciplinary interventions that may be used after phenotype-based assessment.

**Figure 3:**
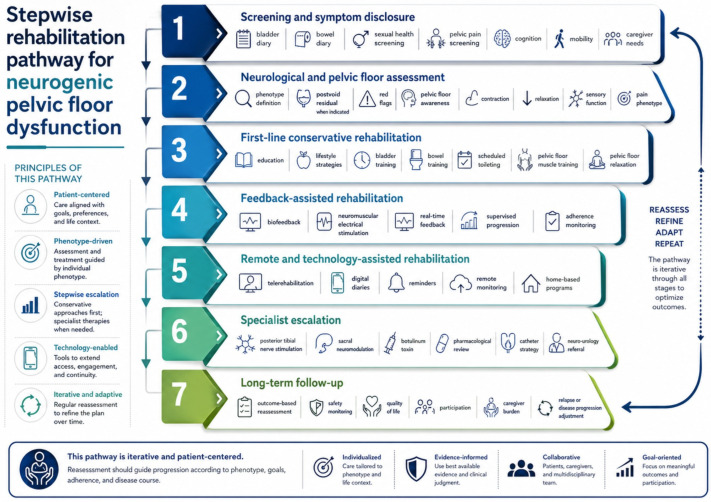
Stepwise rehabilitation pathway for neurogenic pelvic floor dysfunction. Legend: The figure was designed by the authors and graphically refined using the latest available version (5.5) of ChatGPT (OpenAI).

This stepwise pathway illustrates a progressive and iterative model of neurogenic pelvic floor rehabilitation, moving from symptom disclosure and phenotyping to conservative rehabilitation, feedback-assisted therapy, remote or technology-supported care, specialist escalation, and long-term follow-up (see Figure 3). The model emphasizes that progression should be guided by symptom phenotype, safety profile, patient goals, adherence, and disease course rather than by diagnosis alone.

The rehabilitation message is therefore nuanced. PFMT is important, but it is not sufficient for every patient. Behavioral training, biofeedback, NMES, telerehabilitation, neuromodulation, and specialist referral can become relevant at different points in a stepwise pathway. The clinical priority is to match the least burdensome plausible intervention to the dominant phenotype, while monitoring safety and participation.

The practical delivery of PFMT in neurological populations requires more than prescribing contractions. The clinician should determine whether the patient can perceive the pelvic floor, isolate contraction without excessive substitution, relax after contraction, repeat the task without provoking pain or urgency, and integrate the skill into transfers, coughing, or urgency suppression when appropriate. Patients with pain, high resting tone, or dyssynergy voiding may need breathing, relaxation, and coordination before strengthening becomes acceptable. For pain-dominant or overactivity-dominant phenotypes, treatment may need to begin with down-training, graded exposure, breathing coordination, and relaxation before strengthening is introduced.

Biofeedback and electrical stimulation can be useful when verbal instruction is insufficient, but their value depends on the target. Visual or auditory feedback may support motor learning when proprioception, sensation, attention, or confidence are impaired. NMES may assist activation in selected patients or modulate afferent pathways, although parameters, electrode placement, session frequency, and maintenance schedules vary widely. Probe-based approaches may be inappropriate in severe pain, sensory intolerance, infection risk, lack of consent capacity, or anatomical barriers.

Telerehabilitation can improve access, privacy, and continuity, but it should be selected rather than assumed. A safe remote program may include baseline screening, symptom diaries, adapted instruction, feedback when feasible, adverse-event instructions, adherence monitoring, and escalation criteria. The therapeutic ladder remains flexible. Education and diaries may be sufficient for mild symptoms, while refractory symptoms, high-risk storage, retention, autonomic dysreflexia, recurrent infection, or severe constipation may require specialist neuro-urological management.

## 6. Toward Precision Neurorehabilitation for Pelvic Floor Dysfunction

### 6.1. Patient Selection, Phenotyping, Adherence, Cognitive Impairment, Caregiver Involvement, and Outcome Measurement

Precision neurorehabilitation begins with patient selection. The first question is not whether PFMT, biofeedback, NMES, PTNS, or SNM are generally effective. The first question is whether the patient’s dominant phenotype, neurological capacity, safety profile, and goals match the mechanism of the intervention. A patient with MS and preserved pelvic floor awareness may benefit from progressive PFMT and urgency suppression. A patient with stroke and severe aphasia may require simplified cues, caregiver involvement, and environmental toileting plans before isolated PFMT becomes realistic. A patient with SCI and high-pressure storage dysfunction needs renal risk management before QoL-focused training is prioritized. A patient with fibromyalgia and pelvic pain may need down-training, graded exposure, and pain education rather than maximal strengthening.

**Figure 4:**
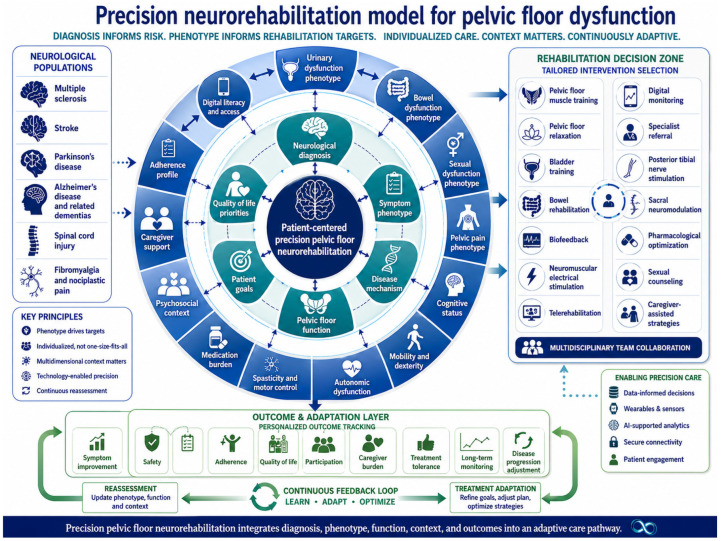
Precision neurorehabilitation model for pelvic floor dysfunction. Legend: The figure was designed by the authors and graphically refined using the latest available version (5.5) of ChatGPT (OpenAI).

Advanced neuro-urological interventions also require precision selection. Sacral neuromodulation has been evaluated for NLUTD, and the literature suggests benefits in selected patients, although responder profiles and long-term durability require careful interpretation [113,114]. Sacral nerve stimulation in PD with OAB symptoms has shown potential, but patients with progressive neurodegeneration require realistic counseling about disease progression and device expectations [115]. Robotic and exoskeleton-assisted gait training introduces another dimension. A randomized pilot trial reported that exoskeleton gait training may improve lower urinary tract function in people with motor-complete SCI, suggesting that locomotor, autonomic, and pelvic organ rehabilitation may interact [116]. Figure 4 presents a precision neurorehabilitation model that integrates phenotype, disease mechanism, rehabilitation target, adherence profile, caregiver support, digital monitoring, and outcome-based follow-up.

This conceptual model illustrates a patient-centered precision approach to pelvic floor neurorehabilitation. Neurological diagnosis informs the risk profile, whereas symptom phenotype, disease mechanism, pelvic floor function, patient goals, quality-of-life priorities, functional status, adherence, caregiver support, psychosocial context, and digital access interact to guide tailored intervention selection. Outcomes are monitored through symptom improvement, safety, adherence, quality of life, participation, caregiver burden, treatment tolerance, long-term monitoring, and disease progression adjustment, creating an iterative feedback loop for reassessment and treatment adaptation.

The proposed precision framework should be interpreted as a clinical reasoning model rather than a validated decision algorithm. Its value lies in organizing patient-specific mechanisms, phenotypes, feasibility factors, and outcomes that future studies can test prospectively.

Adherence is not a secondary issue. PFMT and bladder training depend on repetition, correct technique, feedback, and symptom monitoring. Cognitive impairment can reduce the ability to remember exercises, identify urgency, follow voiding schedules, or report adverse symptoms. Fatigue may limit practice in MS. Bradykinesia, and apathy may affect PD. Aphasia, neglect, or executive dysfunction may affect stroke. Dementia requires caregiver-mediated routines and environmental modification. Chronic pain and fear avoidance may affect fibromyalgia. Precision care therefore includes adherence to phenotyping. The clinician should ask whether the patient can learn the task, perform it safely, repeat it consistently, integrate it into daily life, and recognize when the strategy is not working.

Bladder and bowel training deserve more attention in neurorehabilitation pathways. Cochrane evidence on bladder training frames structured behavioral treatment as more than informal advice [117]. This distinction matters because timed voiding, urge suppression, fluid management, constipation routines, rectal stimulation, medication timing, mobility support, and caregiver prompts require protocolization. Non-neurogenic female LUTS guidelines also emphasize diagnostic clarity and conservative management, reinforcing principles that can be adapted cautiously to neurological populations when risk is low and symptoms are compatible [118]. Evidence discussed above supports the idea that conservative, pharmacological, and minimally invasive therapies should be viewed as complementary steps rather than competing silos.

### 6.2. Multidisciplinary Care Models and Integration into Neurological Rehabilitation Pathways

Medication decisions should be interpreted within rehabilitation goals. Pharmacological treatment may be relevant in selected patients with neurogenic lower urinary tract dysfunction, but it should be considered alongside cognition, constipation, xerostomia, falls risk, alertness, and polypharmacy. Anticholinergic burden is particularly relevant in Parkinson’s disease, dementia, and older stroke survivors, where cognitive decline, constipation, and reduced alertness may undermine rehabilitation participation. These considerations do not imply that medication should be avoided, but that it should be integrated with behavioral and physical rehabilitation and reviewed when it negatively affects cognition, bowel function, or participation.

In patients with suspected high-risk neurogenic lower urinary tract dysfunction, rehabilitation planning should be integrated with neuro-urological risk stratification, since quality-of-life-oriented goals cannot replace renal safety, pressure management, and appropriate bladder drainage.

Outcome measurement remains a major weakness of the field. Trials use heterogeneous bladder diaries, pad tests, quality-of-life questionnaires, pelvic floor muscle scales, urodynamic endpoints, bowel measures, sexual outcomes, and adherence indicators. The Qualiveen questionnaire, used to assess urinary quality of life in MS, remains an example of disease-relevant symptom impact assessment [119]. However, broader harmonization is still needed across spinal cord injury datasets, bowel instruments, sexual health scales, participation outcomes, caregiver measures, and feasibility indicators. For future clinical trials and implementation studies, a minimum core outcome framework should include urinary symptoms, neurogenic safety, bowel function, sexual function, pelvic pain, quality of life, participation, adherence, feasibility, caregiver burden, adverse events, and health-care utilization. Urinary outcomes may include a bladder diary, ICIQ-UI SF, OAB-q, pad test, postvoid residual, and urodynamics when clinically indicated. Bowel outcomes may include the Neurogenic Bowel Dysfunction score, bowel diary, bowel care time, constipation severity, and fecal incontinence episodes. Sexual outcomes may include the Female Sexual Function Index, International Index of Erectile Function, sexual quality-of-life scales, and structured clinical interview. Pelvic pain outcomes may include numerical rating scales, pain diagrams, pelvic floor examination findings, and central sensitization measures when relevant. Caregiver-related outcomes should include validated burden measures such as the Zarit Burden Interview or Caregiver Strain Index, together with care-time logs when appropriate. Follow-up should be standardized whenever possible, with assessment at baseline, immediately after treatment, short-term follow-up at 6–12 weeks or approximately 3 months, medium-term follow-up at 6 months, and long-term follow-up at 12 months when feasible. This structure would improve comparability across neurological populations and support future meta-analysis. Table 3 outlines recommended outcome domains and assessment tools.

A precision pathway requires multidisciplinary ownership. Neurologists may identify disease trajectory and medication interactions; physiatrists may integrate bladder, bowel, sexual, and pelvic symptoms into functional rehabilitation; urologists and neuro-urologists may manage risk stratification, urodynamics, pharmacotherapy, botulinum toxin, catheter strategies, and neuromodulation; pelvic floor physiotherapists may deliver pelvic floor muscle training, down-training, biofeedback, and behavioral interventions; nurses often coordinate bladder and bowel routines; psychologists and sexologists may address avoidance, depression, trauma, body image, relationship concerns, and sexual adaptation; and caregivers may support adherence in daily routines.

The practical goal is not to create a separate pelvic floor pathway isolated from neurological rehabilitation, but to embed pelvic floor assessment and treatment into existing care pathways. Screening questions can be included in neurological intake forms, bladder and bowel diaries can be used when symptoms are present, and pelvic floor assessment can be offered when leakage, constipation, sexual dysfunction, or pelvic pain interferes with rehabilitation goals. Referral criteria should identify high-risk neurogenic lower urinary tract dysfunction, recurrent infection, urinary retention, hematuria, severe constipation, autonomic dysreflexia, and refractory symptoms.

Precision neurorehabilitation also requires a temporal model. During acute hospitalization, scheduled toileting, transfer assistance, medication review, bladder scanning, renal safety, catheter routines, and bowel programs may be more realistic than intensive isolated pelvic floor muscle training. During later rehabilitation and community reintegration, pelvic floor muscle training, urgency suppression, confidence building, sexuality-related goals, technology-assisted training, and participation-oriented interventions may become more feasible. Pelvic floor goals should therefore be reassessed over time rather than treated as fixed.

Patient selection should consider the ability to participate, but exclusion should not become neglect. Patients with dementia, aphasia, severe fatigue, or mobility dependence often need pelvic floor-related care precisely because they cannot manage symptoms independently. Adapted protocols may use simplified instructions, environmental cues, caregiver prompts, scheduled routines, short practice sessions, and simplified diaries. In these cases, meaningful outcomes may include fewer accidents, less distress, better skin protection, reduced care time, safer transfers, improved sleep, or greater dignity, even when standard exercise-based endpoints are unrealistic.

Implementation should also consider equity and scalability. Specialized pelvic floor physiotherapy, neuro-urology, sacral neuromodulation, posterior tibial nerve stimulation, robotics, and telerehabilitation are not equally available across health systems. A precision pathway should therefore be clinically sophisticated but also feasible in low-resource settings. Screening tools, basic diaries, first-line education, caregiver training, referral criteria, and standardized outcome forms can be implemented even where advanced technologies are unavailable.

Finally, outcome-based follow-up is essential. Each intervention should begin with a defined target, such as fewer urgency leaks, shorter bowel care time, improved sexual comfort, reduced pelvic pain, safer catheter routines, improved sleep, fewer therapy interruptions, or reduced caregiver burden. Follow-up should assess whether the target changed, whether adverse effects occurred, whether the patient adhered, and whether the goal remains relevant. Outcome selection should reflect both organ-specific safety and patient-centered function, because a treatment that reduces symptoms but increases dependence, anxiety, or care complexity may not represent meaningful rehabilitation for the patient or family.

## 7. Discussion

### 7.1. Critical Interpretation of the Evidence, Clinical Implications, and Unresolved Questions

Should PFD be considered only a secondary complication in neurological disorders? Available evidence suggests that this framing may be too narrow. Urinary, bowel, sexual, and pain symptoms can interact with mobility, participation, mood, caregiver burden, and adherence to rehabilitation. A patient who avoids therapy because of leakage, constipation, pelvic pain, or fear of accidents is experiencing a functional barrier, not only a peripheral symptom. Routine screening may therefore help rehabilitation teams identify treatable contributors to disability that might otherwise remain hidden.

A second question is whether pelvic floor rehabilitation should be disease-specific or phenotype-specific. The most defensible answer is both. Disease-specific knowledge helps anticipate mechanisms, risks, and feasibility barriers. MS raises questions about fatigue, relapses, sensory impairment, and spinal demyelination. Stroke raises questions about behavior control, aphasia, neglect, and mobility. PD raises questions about autonomic dysfunction, constipation, cognition, falls, and anticholinergic burden. Dementia raises questions about functional incontinence, environmental cues, and caregiver-mediated routines. SCI raises questions about lesion level, autonomic dysreflexia, bowel management, sexuality, and renal risk. Phenotype-specific assessment then determines whether the rehabilitation target is urgency inhibition, pelvic floor coordination, relaxation, bowel predictability, sexual adaptation, pain reduction, or specialist escalation.

A third question concerns the position of PFMT in conservative care. PFMT can remain a central tool when the patient can identify, contract, relax, and train the pelvic floor muscles with sufficient repetition. It may be incomplete when urgency is driven mainly by supraspinal disinhibition, when retention reflects impaired detrusor contractility, when constipation reflects slow transit or dependent toileting, or when pelvic pain reflects overactivity and central sensitization. A cautious therapeutic ladder therefore includes education, bladder training, bowel training, pelvic floor relaxation, biofeedback, NMES, telerehabilitation, neuromodulation, and neuro-urology referral when indicated.

Clinical ownership should also be shared. Neurogenic PFD sits at the intersection of neurology, urology, rehabilitation, nursing, pelvic floor physiotherapy, psychology, and sexual medicine. A single-discipline model may miss either the neurological mechanism, the pelvic floor target, the renal risk, or the behavioral context. Multidisciplinary care is more complex, but it better reflects the lived experience of patients who do not separate bladder, bowel, sexual function, pain, mobility, and dignity into different compartments.

The current evidence remains uneven. Many trials are small, single-center, short-term, and heterogeneous in intervention dose. Control conditions vary, and sham procedures are not always convincing. Outcome measures differ across studies, which limits cross-disease comparison. Neurological populations are often selected for ability to participate, which may overestimate feasibility in patients with severe cognitive, sensory, communication, or mobility impairments. Bowel, sexual, pelvic pain, and caregiver outcomes remain less represented than urinary outcomes. These limitations justify cautious interpretation and argue against broad claims that any single intervention is effective for all neurogenic PFD phenotypes. To make this uncertainty more explicit for clinical readers, Table 2 now includes simplified narrative evidence-strength descriptors (high, moderate, low, very low, or exploratory). These descriptors are clinically interpretive and were not derived from a formal GRADE assessment, which would be inappropriate for the present narrative design.

### 7.2. Strengths and Limitations of the Current Evidence and Future Directions

The strengths of this narrative review include its cross-disease scope, its integration of neuro-urological mechanisms with rehabilitation targets, and its emphasis on clinical phenotyping. The review also highlights topics that are often under-discussed, including dementia, fibromyalgia, sexual function, caregiver involvement, and digital delivery. Its limitations derive from narrative design. It does not provide pooled effect estimates, risk-of-bias ratings, or formal certainty of grading. The literature is also evolving quickly, and new trials may refine the pathway proposed here. A further limitation is that PubMed was the only database consulted, which may have restricted the retrieval of relevant studies indexed exclusively elsewhere and may therefore have influenced the breadth of our findings. Nevertheless, the purpose of this narrative review was to provide a clinically oriented overview rather than an exhaustive systematic synthesis. PubMed was selected because it was considered the most appropriate biomedical database for the clinical and translational scope of this narrative review, particularly for neurological, neuro-urological, rehabilitation, and pelvic floor research. In line with the narrative design of the review, no formal risk-of-bias or certainty-of-evidence assessment was performed.

Future research should move beyond single-symptom trials. A study that measures only urinary frequency may miss outcomes that matter to patients, such as confidence leaving home, reduced caregiver burden, improved sleep, sexual participation, bowel independence, or fewer therapy interruptions. Core outcome sets are needed for neurogenic PFD. They should include urinary, bowel, sexual, pain, QoL, participation, adherence, caregiver, safety, and health-economic domains. Disease-specific modules may still be useful, but a shared outcome architecture would improve comparison across trials.

Future trials should also use phenotype-based inclusion criteria. Enrolling all patients with a broad diagnosis such as MS or stroke may combine different mechanisms and dilute treatment effects. Disease-informed phenotype trials may provide a more balanced design. An MS trial could stratify by urgency-predominant LUTS, pelvic floor awareness, fatigue, and disability. A PD trial could stratify by OAB symptoms, constipation, cognition, and fall risk. A SCI trial could stratify by lesion completeness, urodynamic risk, bowel routine burden, and residual pelvic floor activation.

Digital health and telerehabilitation deserve careful development rather than indiscriminate expansion. Remote care can improve access, privacy, and continuity, but it may exclude patients with cognitive impairment, poor internet access, severe disability, or low digital confidence. Hybrid models may be more realistic, with initial in-person phenotyping followed by supervised remote progression, electronic diaries, video feedback, and escalation pathways. Digital monitoring should support clinician reasoning and patient autonomy rather than replace individualized assessment.

The field should also examine interactions between motor rehabilitation and pelvic organ function. Robotics, exoskeleton gait training, non-invasive spinal stimulation, and task-oriented mobility programs may influence autonomic, sensory, and pelvic floor circuits. These effects should be measured intentionally, especially in SCI and stroke, where locomotor and pelvic organ circuits may share spinal and supraspinal modulation. Table 4 summarizes priority research gaps and future directions for precision pelvic floor neurorehabilitation.

A final question is how this review differs from recent focused summaries of neurogenic PFD. This review uses a broader precision rehabilitation frame and emphasizes disease-informed phenotyping, intervention matching, outcome standardization, and integration into neurorehabilitation clinical pathways. This emphasis is relevant because focused reviews have already highlighted clinical relevance, while a subsequent step is to translate that relevance into reproducible pathways that can be tested, audited, and implemented [13].

Implementation of science will be important for the next phase of this field. A strong pathway may have limited impact unless clinical teams know when to screen, how to document symptoms, when to refer, and how to communicate pelvic floor goals without increasing stigma. Training packages for neurological rehabilitation teams may include pelvic floor terminology, red flags for neuro-urology referral, safe use of bladder and bowel diaries, recognition of pelvic pain overactivity or guarding, and structured approaches to sexual health discussion.

Service models should also define responsibility. A nurse-led screen may identify symptoms, a physiotherapist may phenotype pelvic floor performance, a physiatrist may integrate goals into rehabilitation, and a urologist or neuro-urologist may manage high-risk NLUTD. Future studies should therefore test not only whether an intervention works, but whether it can be delivered consistently by real teams in real services. Cost, training time, patient acceptability, digital access, privacy, and referral capacity will shape implementation as strongly as physiological efficacy.

Several questions remain open. It is uncertain which neurological phenotypes respond best to supervised PFMT, which patients benefit most from biofeedback or NMES, and how long gains persist after treatment ends. It is also unclear how digital monitoring should be adapted for patients with cognitive impairment or caregiver dependence. These uncertainties do not weaken the rationale for rehabilitation. They indicate where rigorous, pragmatic, and patient-centered studies are most needed. Trial design may also need to include patients who are commonly excluded from highly controlled protocols, including those with fatigue, aphasia, cognitive impairment, caregiver dependence, or complex mobility needs, because these groups often represent the realities of neurological rehabilitation services.

## 8. Conclusions

Pelvic floor dysfunction in neurological disorders should be conceptualized as a multidimensional neurorehabilitation target rather than solely as a urological or gynecological comorbidity. Bladder, bowel, sexual, and pelvic pain symptoms can substantially affect participation, dignity, mood, caregiver burden, adherence, and functional recovery. Their mechanisms are heterogeneous, involving cortical, brainstem, spinal, autonomic, sensory, cognitive, and mobility-related pathways.

A clinically useful approach requires phenotype recognition and individualized treatment matching. The same neurological diagnosis may be associated with urgency, leakage, retention, constipation, fecal incontinence, dyspareunia, pelvic pain, cognitive barriers, caregiver dependence, or combined pelvic organ dysfunction. Interventions such as pelvic floor muscle training, bladder and bowel training, biofeedback, neuromuscular electrical stimulation, tibial nerve stimulation, sacral neuromodulation, telerehabilitation, robotics, and sexual counseling may each have a role, but their value depends on the underlying mechanism, safety profile, patient goals, and rehabilitation context.

Future research should develop phenotype-specific, disease-informed, outcome-standardized, and implementation-ready pathways for precision pelvic floor neurorehabilitation. Larger trials and pragmatic implementation studies should integrate urinary, bowel, sexual, pain, quality-of-life, participation, adherence, safety, and caregiver outcomes. Embedding pelvic floor screening and referral pathways into neurological rehabilitation may help address these symptoms earlier, more systematically, and more respectfully, while improving shared decision-making and documenting outcomes that matter in daily life.

## Figures and Tables

**Table 1:** Disease-specific phenotypes of pelvic floor dysfunction in neurological disorders.

Neurological Condition	Dominant Neurological Mechanism	Main Urinary Phenotype	Bowel Involvement	Sexual and Pelvic Pain Involvement	Assessment Priorities	Rehabilitation Implications
Multiple sclerosis	Demyelinating lesions affecting supraspinal and spinal storage or voiding pathways	Urgency, frequency, urgency UI, mixed LUTS, detrusor overactivity, possible dyssynergia [36,37,38,39,40]	Constipation and evacuation difficulty may coexist with fatigue, immobility, or autonomic dysfunction	Sexual dysfunction, reduced arousal, dyspareunia, sensory symptoms, and pelvic pain may coexist	Bladder diary, PVR, symptom burden, pelvic floor awareness, fatigue, cognition, urodynamics when risk or refractory symptoms exist	PFMT, urgency suppression, biofeedback, NMES, telerehabilitation, PTNS, and escalation to neuro-urology when high-risk LUTD is suspected
Stroke	Suprapontine injury, behavior control deficits, sensory-motor impairment, mobility limitation	UI, urgency, OAB-like symptoms, acute retention, elevated PVR in selected patients [41,42,43,44,45,46,47]	Constipation may reflect immobility, dysphagia-related diet change, medication, or dependent toileting	Reduced libido, arousal difficulty, dyspareunia, fear, altered self-image, and pelvic pain can affect participation	Cognition, aphasia, neglect, sensory awareness, transfers, PVR, continence trajectory, caregiver support	PFMT when contraction awareness is preserved, bladder training, scheduled toileting, simplified cues, caregiver-assisted routines
Parkinson’s disease	Basal ganglia and dopaminergic disruption with autonomic and cognitive involvement	Urgency, frequency, nocturia, OAB, UI, possible retention in advanced or atypical syndromes [48,49,50,51,52,53,54]	Constipation is common and may worsen urinary urgency and medication tolerance	Erectile dysfunction, reduced desire, autonomic sexual dysfunction, and relationship concerns	Medication review, constipation, falls, cognition, orthostatic symptoms, bladder diary, PVR when indicated	Behavioral therapy, bladder training, pelvic floor strategies, cautious pharmacotherapy, PTNS or SNM in selected refractory cases
Alzheimer’s disease and related dementias	Executive dysfunction, impaired awareness, apraxia, mobility limitation, environmental barriers, possible LUT pathology	Functional UI, urgency, nocturia, incontinence with limited symptom reporting [55,56,57,58,59,60]	Constipation related to immobility, hydration, diet, and medication burden	Sexual health is rarely assessed but may be affected by cognition, consent, intimacy, and caregiver context	Functional assessment, toileting access, caregiver capacity, medication burden, reversible triggers, PVR when clinically indicated	Prompted voiding, scheduled toileting, environmental modification, caregiver training, simplified PFMT only when feasible
Spinal cord injury	Interruption of supraspinal-spinal-sacral control with somatic and autonomic disruption	Detrusor overactivity, detrusor-sphincter dyssynergia, retention, catheter dependence, renal risk [61,62]	Neurogenic bowel dysfunction, constipation, fecal incontinence, prolonged routines, autonomic dysreflexia risk [63,64]	Sexual dysfunction, genital sensory change, fertility concerns, pelvic pain, relationship impact [65,66]	Neurological level and completeness, urodynamics, renal surveillance, bowel routine, autonomic dysreflexia, sexual priorities	Risk-stratified neuro-urology, bowel programs, PFMT if incomplete pathways remain, neuromodulation, robotics, sexual rehabilitation
Fibromyalgia and nociplastic pain	Altered central pain processing, possible pelvic floor overactivity or guarding, sensory amplification, psychological and sleep interactions	Urinary distress, frequency, urgency, bladder pain overlap, pelvic floor symptom burden [67,68,69,70,71,72,73]	IBS-like symptoms, constipation, defecatory dysfunction, pelvic floor dyssynergia in selected patients	Dyspareunia, pelvic myofascial pain, reduced desire, sexual dissatisfaction, pain amplification	Pain phenotype, pelvic floor tone, trauma history, sleep, mood, activity tolerance, central sensitization features	Education, down-training, graded exposure, relaxation, gentle coordination, multidisciplinary pain and sexual counseling

Legend: LUTD, lower urinary tract dysfunction; LUTS, lower urinary tract symptoms; NMES, neuromuscular electrical stimulation; OAB, overactive bladder; PD, Parkinson’s disease; PFMT, pelvic floor muscle training; PTNS, posterior tibial nerve stimulation; PVR, postvoid residual; UI, urinary incontinence.

**Table 2:** Rehabilitation strategies for pelvic floor dysfunction in neurological populations.

Intervention	Primary Target	Mechanism of Action	Candidate Populations	Relevant Outcomes	Evidence Strength (Narrative Descriptor)	Practical Limitations
PFMT	Pelvic floor strength, endurance, coordination, timing	Motor learning and improved urethral or anal closure support	Direct: non-neurogenic female stress/urgency/mixed UI; neurological direct/near-direct: MS and selected stroke; conditional/extrapolated: incomplete SCI with preserved pathways [77,78,79,80,81,82,83]	Leakage episodes, pad use, pelvic floor strength, QoL	High for non-neurogenic female UI; moderate/low for MS and selected stroke; low/extrapolated for other neurological phenotypes	Requires awareness, cognition, repetition, correct technique
Pelvic floor relaxation and down-training	Pelvic pain, overactivity or guarding, dyssynergic voiding or defecation	Reduction of resting tone, improved relaxation, graded exposure, pain-informed motor control	Fibromyalgia and nociplastic pain, pelvic pain overlap, selected MS, stroke, or SCI patients with overactivity or guarding	Pain intensity, dyspareunia, relaxation ability, voiding or defecatory coordination, QoL	Low; clinically plausible for nociplastic or pain-dominant phenotypes	Requires skilled pelvic floor assessment and may need to precede strengthening when pain or overactivity dominates
Bladder training and urgency suppression	Urgency, frequency, OAB behaviors	Gradual voiding interval extension and inhibition of urgency response	Direct: PD behavioral trials; indirect/extrapolated: MS, stroke, dementia with caregiver support [92,93]	Voiding frequency, urgency episodes, nocturia, confidence	Moderate for OAB and PD behavioral trials; low for dementia/caregiver-mediated neurological use	Difficult with cognitive impairment or severe mobility limitation
Bowel training	Constipation, fecal incontinence, unpredictable evacuation	Scheduled evacuation, gastrocolic reflex use, stool consistency and routine optimization	SCI, MS, PD, dementia [63,64]	Bowel care time, accidents, independence, QoL	Low-to-moderate clinical consensus/established practice; heterogeneous trial evidence	Requires individualized routines and caregiver participation in dependent patients
Biofeedback	Awareness and selective pelvic floor activation or relaxation	Visual or auditory reinforcement of hidden muscle activity	MS, stroke, pelvic pain, selected PD and SCI [88]	Contraction quality, coordination, symptom control	Low-to-moderate; heterogeneous and population-dependent	Needs trained therapist, equipment, tolerance of probes or sensors
NMES and electrical stimulation	Muscle activation, sensory input, inhibitory reflexes	Electrical activation of pelvic floor or peripheral afferent pathways	MS, stroke, incomplete SCI, urgency UI [89,94,95,96,97,98,99,100]	Leakage, urgency, muscle strength, urodynamics, QoL	Low-to-moderate; protocol-dependent	Contraindications, discomfort, access, variable parameters
PTNS and tibial stimulation	Storage symptoms and urgency	Peripheral neuromodulation of sacral afferent pathways	MS, PD, selected NLUTD [107,108,109,110,111]	Urgency, frequency, nocturia, OAB scores	Moderate emerging evidence for MS/PD storage symptoms	Repeated sessions, maintenance burden, response variability
SNM	Refractory storage or voiding dysfunction	Chronic modulation of sacral neural circuits	Selected NLUTD, PD, incomplete lesions	Symptom response, QoL, diary outcomes	Moderate in selected refractory NLUTD; careful selection required	Surgical device, cost, disease progression, careful selection required
Telerehabilitation	Access, adherence, continuity	Remote supervision, digital feedback, diaries, progression	Direct: MS and non-neurogenic UI; extrapolated/feasibility-based: PD, SCI, dementia and other mobility-limited patients [79,84,85,86]	Adherence, symptom scores, QoL, access	Moderate in MS/non-neurogenic UI; low/extrapolated for PD, SCI, dementia	Digital literacy, privacy, safety, assessment constraints
Robotics or exoskeleton-assisted training	Locomotor-autonomic interaction	Task-specific gait and spinal network activation	SCI and possibly stroke	LUT function, mobility, autonomy	Exploratory	Cost, access, patient selection, unclear mechanism
Multidisciplinary sexual counseling	Sexual function, intimacy, body image, pelvic pain	Education, adaptation, communication, pelvic floor and psychological integration	SCI, MS, stroke, PD, fibromyalgia [65,66]	Sexual QoL, relationship satisfaction, participation	Low but clinically important and under-studied	Stigma, clinician discomfort, limited referral pathways

Legend: MS, multiple sclerosis; NLUTD, neurogenic lower urinary tract dysfunction; NMES, neuromuscular electrical stimulation; OAB, overactive bladder; PD, Parkinson’s disease; PFMT, pelvic floor muscle training; PTNS, posterior tibial nerve stimulation; QoL, quality of life; SCI, spinal cord injury; SNM, sacral neuromodulation; UI, urinary incontinence. Evidence-strength descriptors are narrative and clinically interpretive; they were not derived from a formal GRADE assessment.

**Table 3:** Recommended outcome domains and assessment tools for neurogenic pelvic floor dysfunction.

Domain	Clinical Construct	Examples of Assessment Tools	Relevant Populations	Recommended Timing	Interpretive Cautions
Urinary symptoms	Urgency, frequency, nocturia, leakage, retention	Bladder diary, ICIQ-UI SF, OAB-q, pad test, PVR, urodynamics	MS, stroke, PD, dementia, SCI	Baseline, post-intervention, follow-up	Symptoms may not match urodynamic mechanism in neurological disease
Neurogenic risk	Upper tract risk and unsafe storage	Urodynamics, renal ultrasound, creatinine when indicated, risk stratification	SCI, MS, high-risk NLUTD	Baseline and risk-based follow-up	QoL-focused outcomes must not replace renal safety assessment
Bowel function	Constipation, fecal incontinence, bowel care burden	Neurogenic Bowel Dysfunction score, bowel diary, bowel care time, accidents [63,64]	SCI, MS, PD, dementia	Baseline and routine review	Objective measures remain less standardized than urinary measures
Sexual function	Desire, arousal, orgasm, erection, dyspareunia, intimacy	FSFI, IIEF, sexual QoL scales, structured interview [65,66]	SCI, MS, stroke, PD, fibromyalgia	Baseline when relevant and after intervention	Consent, cognition, relationship context, and stigma require sensitive assessment
Pelvic pain	Myofascial pain, pudendal neuralgia, bladder pain, nociplastic pain	Pain diagrams, numerical rating scales, pelvic floor palpation, central sensitization measures [30,73]	Fibromyalgia, MS, stroke, pelvic pain overlap	Baseline, during graded progression, follow-up	Strengthening may worsen symptoms if overactivity dominates
QoL and participation	Symptom impact on daily life and rehabilitation	Qualiveen, generic QoL tools, participation scales [119]	All populations	Baseline and post-intervention	Disease-specific QoL tools may miss pelvic floor priorities
Adherence and feasibility	Ability to learn, repeat, and integrate therapy	Exercise logs, digital adherence data, therapist rating, caregiver report	MS, stroke, PD, dementia, SCI	Every visit during intervention	High adherence in trials may not generalize to routine care
Caregiver burden	Support needed for toileting, diaries, exercises, catheter or bowel routines	Zarit Burden Interview, Caregiver Strain Index, care-time logs, structured caregiver interview	Dementia, severe stroke, SCI	Baseline and care-plan reviews	Patient autonomy and caregiver feasibility must be balanced

Legend: FSFI, Female Sexual Function Index; ICIQ-UI SF, International Consultation on Incontinence Questionnaire-Urinary Incontinence Short Form; IIEF, International Index of Erectile Function; MS, multiple sclerosis; NLUTD, neurogenic lower urinary tract dysfunction; OAB-q, Overactive Bladder Questionnaire; PD, Parkinson’s disease; PVR, postvoid residual; QoL, quality of life; SCI, spinal cord injury.

**Table 4:** Research gaps and future directions for precision pelvic floor neurorehabilitation.

Research Area	Current Limitation	Why It Matters	Potential Future Study Design	Priority Outcomes	Clinical Translation Potential
Phenotype-specific PFMT	Broad diagnostic enrollment without precise urinary or pelvic floor phenotype	Treatment effects may be diluted by mixed mechanisms	Multicenter RCTs stratified by urgency, stress leakage, retention, pain, and awareness	Leakage, strength, adherence, QoL, urodynamics when indicated	High if protocols are simple and reproducible
Neurogenic bowel rehabilitation	Fewer trials and inconsistent objective measures	Bowel dysfunction strongly affects independence and QoL	Pragmatic trials of bowel training plus digital or caregiver support	Bowel care time, fecal incontinence, constipation, autonomy	High in SCI, MS, PD, dementia care pathways
Sexual rehabilitation	Under-screening and limited integration with pelvic floor care	Sexual health is a patient priority and affects identity and relationships	Mixed-methods trials integrating pelvic floor, psychological, and sexual counseling	Sexual QoL, dyspareunia, satisfaction, relationship outcomes	High if clinicians receive training and referral pathways exist
Telerehabilitation	Digital exclusion and variable supervision intensity	Remote delivery may improve access but not for all patients	Hybrid comparative trials with digital literacy stratification	Adherence, access, symptom change, safety, satisfaction	High with careful patient selection
Neuromodulation	Heterogeneous stimulation parameters and limited sham-controlled data	Response depends on lesion type and preserved pathways	Disease-specific sham-controlled trials with mechanistic endpoints	Diary outcomes, urodynamics, QoL, adverse events	Moderate to high in refractory phenotypes
Robotic and locomotor-autonomic integration	Pelvic organ outcomes often secondary or exploratory	Motor training may influence autonomic and pelvic circuits	RCTs embedding bladder, bowel, and sexual outcomes into gait trials	LUT function, bowel routines, mobility, participation	Exploratory but promising in SCI and stroke
Cognitive impairment and caregivers	Few protocols are adapted for dementia, aphasia, neglect, or executive dysfunction	Patients with greatest need may be excluded from trials	Caregiver-assisted intervention trials and implementation studies	Continence episodes, caregiver time, dignity, institutionalization risk	High for dementia and severe stroke services
Core outcome sets	Inconsistent measures prevent comparison and meta-analysis	Standardization is needed for trial synthesis and guidelines	Delphi consensus with patients, clinicians, and researchers	Urinary, bowel, sexual, pain, QoL, participation, safety	Very high across the entire field

Legend: LUT, lower urinary tract; MS, multiple sclerosis; PD, Parkinson’s disease; PFMT, pelvic floor muscle training; QoL, quality of life; RCT, randomized controlled trial; SCI, spinal cord injury.

## Data Availability

No new data were created or analyzed in this study. Data sharing is not applicable to this article.

## Supplementary Materials

The following supporting information can be downloaded at: https://www.mdpi.com/article/10.3390/jcm15135140/s1, Table S1: PubMed search strategy and evidence-mapping logic; Table S2: Key evidence by neurological condition; Table S3: Suggested clinical assessment battery for pelvic floor dysfunction in neurorehabilitation.

## Abbreviations

The following abbreviations are used in this manuscript:

AD	Alzheimer’s disease
AUA/SUFU	American Urological Association/Society of Urodynamics, Female Pelvic Medicine & Urogenital Reconstruction
FSFI	Female Sexual Function Index
IBS	Irritable bowel syndrome
ICIQ-UI SF	International Consultation on Incontinence Questionnaire–Urinary Incontinence Short Form
IIEF	International Index of Erectile Function
IRCCS	Istituto di Ricovero e Cura a Carattere Scientifico
LUT	Lower urinary tract
LUTD	Lower urinary tract dysfunction
LUTS	Lower urinary tract symptoms
MS	Multiple sclerosis
NLUTD	Neurogenic lower urinary tract dysfunction
NMES	Neuromuscular electrical stimulation
OAB	Overactive bladder
OAB-q	Overactive Bladder Questionnaire
PD	Parkinson’s disease
PFD	Pelvic floor dysfunction
PFMT	Pelvic floor muscle training
PTNS	Posterior tibial nerve stimulation
PVR	Postvoid residual
QoL	Quality of life
RCT	Randomized controlled trial
SANRA	Scale for the Assessment of Narrative Review Articles
SCI	Spinal cord injury
SNM	Sacral neuromodulation
UI	Urinary incontinence
