# Supplementary material for: Neurogenic Pelvic Floor Dysfunctions Across Neurological Disorders: Mechanisms, Phenotypes, and Precision Rehabilitation Pathways—A Narrative Review
Source: J Clin Med. 2026 Jul 1;15(13):5140. doi: 10.3390/jcm15135140 (PMC13363305; doi:10.3390/jcm15135140)
Supplement: Supplementary file 1 [file jcm-15-05140-s001.zip › jcm-4349939-supplementary.pdf]

Supplementary Materials: Neurogenic Pelvic Floor Dysfunction Across Neurological Disorders

Supplementary Table S1. PubMed search strategy and evidence-mapping logic.

This table provides operational detail for the narrative evidence map. PubMed was used as the primary biomedical source. The search covered database inception to 2 May 2026, with the final search update performed on 2 May 2026. Searches combined MeSH terms and Title/Abstract terms when appropriate. Records were considered when they involved adult human populations and were published in English. Eligible sources included randomized controlled trials, controlled clinical studies, prospective and retrospective observational studies, systematic reviews, meta-analyses, peer-reviewed practice guidelines, terminology reports, and high-quality narrative reviews. Because this was a narrative review rather than a systematic review or meta-analysis, no formal risk-of-bias or certainty-of-evidence assessment was performed.

Concept	Exact PubMed Boolean search string	Evidence priority	Approx. PubMed records retrieved*	Approx. records retained/cited or used in synthesis*	Rationale
General neurogenic pelvic floor dysfunction	("pelvic floor dysfunction"[Title/Abstract] OR "pelvic floor"[Title/Abstract] OR "lower urinary tract symptoms"[Title/Abstract] OR LUTS[Title/Abstract] OR "urinary incontinence"[Title/Abstract] OR "neurogenic bladder"[Title/Abstract] OR "neurogenic lower urinary tract dysfunction"[Title/Abstract] OR NLUTD[Title/Abstract] OR "bowel dysfunction"[Title/Abstract] OR constipation[Title/Abstract] OR "fecal incontinence"[Title/Abstract] OR "sexual dysfunction"[Title/Abstract] OR "pelvic pain"[Title/Abstract]) AND (neurologic*[Title/Abstract] OR neurological[Title/Abstract] OR neurorehabilitation[Title/Abstract] OR "nervous system diseases"[MeSH Terms])	Trials, systematic reviews, clinical studies, guidelines, and clinically relevant narrative reviews	approx. 1,270	approx. 35	Identify rehabilitation and symptom evidence across neurological populations.
Neuro-urology and NLUTD	("neurogenic bladder"[Title/Abstract] OR "neurogenic lower urinary tract dysfunction"[Title/Abstract] OR NLUTD[Title/Abstract] OR "lower urinary tract symptoms"[Title/Abstract] OR "urinary incontinence"[Title/Abstract]) AND (rehabilitation[Title/Abstract] OR biofeedback[Title/Abstract] OR "pelvic floor muscle training"[Title/Abstract] OR neuromodulation[Title/Abstract] OR urodynamics[Title/Abstract])	Guidelines, terminology reports, urodynamic studies, systematic reviews	approx. 620	approx. 28	Map urinary mechanisms, risk stratification, and rehabilitation targets.
Disease-specific neurological populations	("pelvic floor"[Title/Abstract] OR "urinary incontinence"[Title/Abstract] OR "bowel dysfunction"[Title/Abstract] OR constipation[Title/Abstract] OR "sexual dysfunction"[Title/Abstract] OR "pelvic pain"[Title/Abstract]) AND ("multiple sclerosis"[Title/Abstract] OR stroke[Title/Abstract] OR poststroke[Title/Abstract] OR "post-stroke"[Title/Abstract] OR Parkinson*[Title/Abstract] OR Alzheimer*[Title/Abstract] OR dementia[Title/Abstract] OR "spinal cord injury"[Title/Abstract] OR SCI[Title/Abstract])	Disease-specific clinical and rehabilitation evidence	approx. 890	approx. 45	Identify diagnosis-specific phenotypes and feasibility barriers.
Rehabilitation and technology-assisted interventions	("pelvic floor muscle training"[Title/Abstract] OR PFMT[Title/Abstract] OR biofeedback[Title/Abstract] OR "neuromuscular electrical stimulation"[Title/Abstract] OR NMES[Title/Abstract] OR "posterior tibial nerve stimulation"[Title/Abstract] OR PTNS[Title/Abstract] OR "sacral neuromodulation"[Title/Abstract] OR "sacral nerve stimulation"[Title/Abstract] OR telerehabilitation[Title/Abstract] OR "digital health"[Title/Abstract] OR robotics[Title/Abstract] OR exoskeleton[Title/Abstract]) AND ("neurogenic bladder"[Title/Abstract] OR "neurogenic lower urinary tract dysfunction"[Title/Abstract] OR "urinary incontinence"[Title/Abstract] OR "pelvic floor dysfunction"[Title/Abstract])	RCTs, systematic reviews, meta-analyses, feasibility studies, and implementation-relevant technology papers	approx. 540	approx. 40	Map feedback-assisted, neuromodulatory, and remote delivery strategies.

Fibromyalgia, nociplastic pain, pelvic pain, and sexual function	(fibromyalgia[MeSH Terms] OR fibromyalgia[Title/Abstract] OR nociplastic[Title/Abstract] OR "central sensitization"[Title/Abstract] OR "chronic pelvic pain"[Title/Abstract]) AND ("pelvic floor"[Title/Abstract] OR "pelvic pain"[Title/Abstract] OR "bladder pain"[Title/Abstract] OR "urinary symptoms"[Title/Abstract] OR "bowel symptoms"[Title/Abstract] OR "sexual dysfunction"[Title/Abstract])	Mechanistic and clinical studies	approx. 310	approx. 18	Capture adjacent nociplastic pain evidence relevant to pelvic pain, guarding, and sexual symptoms.

*Counts are approximate and rounded because the same article could be retrieved by more than one search string. Because this was a narrative review, these counts are intended to improve transparency of source identification and do not represent a PRISMA-style screening flow. Records could overlap across search strings, and the final included sources were selected according to conceptual relevance, disease specificity, clinical relevance, and contribution to the evidence-mapping framework. Total sources included/cited in the narrative synthesis: 120.

Supplementary Table S2. Key evidence by neurological condition.

This table summarizes why each neurological or adjacent mechanistic population was included in the narrative framework.

Condition	Key evidence types	Most relevant clinical emphasis	Reason for inclusion in this narrative framework
MS	Systematic reviews, RCTs, telerehabilitation trials	Urgency, UI, fatigue, QoL, PFMT, biofeedback, neuromodulation	Demyelinating supraspinal and spinal pathways with urinary, bowel, sexual, and fatigue-related overlap.
Stroke	Clinical surveys, post-stroke LUTS studies, PFMT reviews	UI, behavior control, PVR, cognition, mobility	Suprapontine and functional continence mechanisms with recovery-stage-dependent feasibility.
PD	Guidelines, RCTs, medication and neuromodulation reviews	OAB, constipation, cognitive medication risk, behavioral therapy	Basal ganglia-autonomic dysfunction, constipation, medication vulnerability, and falls risk.
Alzheimer’s disease and related dementias	Cohort, urodynamic, and primary care studies	Functional UI, caregiver-assisted toileting, medication burden	Functional continence, cognition, environmental access, and caregiver dependence.
SCI	Longitudinal cohorts, neuromodulation reviews, bowel studies	Risk stratification, bowel care, sexuality, autonomic safety	Direct neurogenic bladder, bowel, sexual, and autonomic disruption requiring safety planning.
Fibromyalgia and nociplastic pain	Systematic review, cross-sectional studies, pain mechanism studies	Pelvic pain, sexual dysfunction, guarding, nociplastic pain features	Adjacent nociplastic pain model illustrating central pain processing and pelvic floor guarding.

Abbreviations: LUTS, lower urinary tract symptoms; MS, multiple sclerosis; OAB, overactive bladder; PD, Parkinson’s disease; PFMT, pelvic floor muscle training; PVR, postvoid residual; QoL, quality of life; RCT, randomized controlled trial; SCI, spinal cord injury; UI, urinary incontinence.

Supplementary Table S3. Suggested clinical assessment battery for pelvic floor dysfunction in neurorehabilitation.

This table proposes a pragmatic assessment sequence that can be adapted to neurological rehabilitation settings.

Step	Assessment element	Purpose	Escalation trigger
1	Screen for urinary, bowel, sexual, and pelvic pain symptoms	Identify hidden barriers to rehabilitation	Severe distress, recurrent leakage, fecal incontinence, sexual pain, or unreported caregiver burden
2	Record neurological diagnosis, lesion level, cognition, mobility, fatigue, and medications	Define feasibility and safety context	Aphasia, neglect, severe fatigue, autonomic symptoms, polypharmacy, or inability to follow instructions
3	Use bladder and bowel diaries when symptoms are present	Quantify frequency, triggers, and routines	Elevated symptom frequency, nocturia, prolonged bowel care, or unclear leakage pattern
4	Assess pelvic floor awareness, contraction, relaxation, and pain when appropriate	Match PFMT, down-training, or biofeedback	Pain provocation, high tone, absent awareness, dyssynergia, or inability to relax after contraction
5	Measure postvoid residual and consider urodynamics when risk or refractory symptoms exist	Avoid unsafe management of NLUTD	Elevated PVR, suspected unsafe storage, recurrent urinary tract infection, hematuria, autonomic dysreflexia, or renal risk
6	Define patient-centered goals and caregiver role	Improve adherence and relevance	Caregiver overload, inability to perform home program, lack of privacy, or limited digital access
7	Select first-line rehabilitation and escalation criteria	Create a reproducible care pathway	Refractory symptoms, safety concerns, worsening pain, severe constipation, or need for neuro-urology referral

Abbreviations: *NLUTD, neurogenic lower urinary tract dysfunction; PFMT, pelvic floor muscle training; PVR, postvoid residual.*
